# Genomic-island cassette architecture provides interpretable signal for exploratory classification of poultry-associated *Enterococcus cecorum* lineages

**DOI:** 10.3389/fmicb.2026.1882753

**Published:** 2026-07-08

**Authors:** Rushikesh R. Lagad, Shakil Rafi, Aranyak Goswami

**Affiliations:** 1 Department of Animal Science, Center for Agricultural Data Analytics (CADA), University of Arkansas System Division of Agriculture, Fayetteville, AR, United States; 2 Data Science Program (DTSC), University of Arkansas, Fayetteville, AR, United States

**Keywords:** accessory genome, antimicrobial resistance, comparative genomics, genomic epidemiology, horizontal gene transfer, interpretable machine learning, mobile genetic elements, poultry pathogen surveillance

## Abstract

**Background:**

*Enterococcus cecorum* is an emerging poultry pathogen whose antimicrobial resistance and host-associated traits are often carried on genomic islands. Standard comparative genomics workflows usually reduce genomes to unordered gene inventories and may miss informative neighborhood structure within island-associated modules.

**Methods:**

We tested whether GI (genomic island)-anchored cassette organization provides signal for distinguishing pathogenic from commensal poultry-associated *E. cecorum* lineages. We encoded genomic-island-anchored cassette organization as 84 genome-level summary features and evaluated this representation in 145 genomes (95 commensal, 50 pathogenic) using locked 5-fold genome-grouped cross-validation.

**Results:**

The cassette-summary Random Forest model achieved an area under the receiver operating characteristic curve (AUROC) of 0.918 ± 0.067, outperforming GI burden (AUROC 0.791 ± 0.050) and assembly-quality (AUROC 0.743 ± 0.015) baselines and performing similarly to a corrected AMR gene-content baseline (AUROC 0.906 ± 0.044). A conservative GI-restricted gene product presence/absence proxy achieved AUROC 0.887 ± 0.083, while a full joint-run pangenome GPA baseline remains a necessary future benchmark. Fragmentation-controlled analyses confirmed cassette signal remained informative after quality filtering (AUROC 0.827 in assemblies with ≤50 contigs; *n* = 91), while leave-one-BioProject-out validation yielded AUROC 0.694, indicating that deployment in novel surveillance contexts requires prospective validation. SHapley Additive exPlanations (SHAP) analysis localized discriminant signal to GI-anchored modules enriched for AMR cargo, mobility load, and GI AMR density.

**Conclusion:**

These results suggest that cassette architecture captures signal consistent with biologically meaningful genomic organization beyond bulk island burden and supports its use as an interpretable exploratory representation for surveillance-oriented analysis of poultry-associated *E. cecorum*, while prospective validation in independent surveillance collections and a full joint-run pangenome gene presence/absence benchmark remain necessary before operational deployment claims can be made.

## Introduction

*Enterococcus cecorum* (EC) has emerged as an important poultry-associated pathogen with outbreaks linked to osteomyelitis, spondylitis, lameness, early-life sepsis, and significant production losses (Higuita et al., 2025; Jung et al., 2018; Robbins et al., 2012; De Herdt et al., 2009; Borst et al., 2017; Jung and Rautenschlein, 2014; Grund et al., 2021; Rhoads et al., 2024). Molecular epidemiology documents multiple circulating EC lineages and outbreak-associated clones (Borst et al., 2012; Boerlin et al., 2012; Wijetunge et al., 2012; Dolka et al., 2016), and comparative genomics reveals extensive accessory genome variation including intercontinental spread of pathogenic lineages and genomic heterogeneity among sepsis-associated broiler isolates (Laurentie et al., 2023; Borst et al., 2015; Souillard et al., 2022; Rhoads et al., 2024). Many clinically relevant traits are encoded on mobile genetic elements particularly genomic islands (GIs) which package resistance, virulence, and mobility functions into transferable modules (Borst et al., 2015; Jackson et al., 2015; Sharma et al., 2020; Huang et al., 2024; Siguier et al., 2014). Standard gene-presence workflows treat these loci as independent features, losing information about neighborhood structure, GI context, and the physical co-organization that characterizes horizontally acquired modules.

Standard genome comparison approaches reduce genomes to three types of summary that discard neighborhood structure. Gene presence/absence (GPA) frameworks treat genomes as unordered ortholog inventories, discarding physical adjacency and GI co-localisation. AMR-burden approaches summarize resistance gene counts or classes, conflating cargo identity with genomic organization. Assembly-quality proxies (e.g., contig count) capture fragmentation differences but contain no biological specificity. None of these representations preserves the higher-order architecture of how genes are co-organized within GI-anchored cassette modules. We therefore benchmarked cassette features against AMR gene content, GI-burden summaries, assembly-quality proxies, and a conservative GI-restricted gene product presence/absence proxy. A full joint-run PIRATE GPA matrix was not available for this submission because the available commensal and pathogenic PIRATE outputs were generated in separate runs and therefore do not provide directly comparable ortholog-family definitions across classes; a unified joint pangenome run remains an important next benchmark.

In EC, pathogenic lineages appear to carry recurrent co-organizations of AMR cargo, mobility functions, and host-adaptation genes within GI-anchored modules that are acquired and transmitted as physically linked blocks during horizontal gene transfer (Borst et al., 2015; Siguier et al., 2014). Prior comparative studies suggest that insertion-sequence-associated rearrangements, plasmid replication functions, and CRISPR-related loci may recur in pathogenic genomes, consistent with modular rather than locus-by-locus acquisition. This modular architecture motivates a structural-genomics view: predictive signal for pathogenic lineage classification may reside in cassette neighborhood co-organization, not just gene inventory.

Here, we tested the hypothesis that genomic-island-anchored cassette organization contains predictive signal for pathogenic lineage classification in poultry-associated *E. cecorum*. To evaluate this, we encoded cassette organization as a genome-level representation and benchmarked it against genomic-island burden, AMR gene content, and assembly-quality baselines under leakage-safe evaluation. We further asked whether the resulting signal remained informative after fragmentation control and cross-project validation, and whether model explanations could localize discriminant signal to interpretable GI-associated modules.

## Materials and methods

### Genome cohort, phenotype labels, and annotation

**FIGURE 1:**
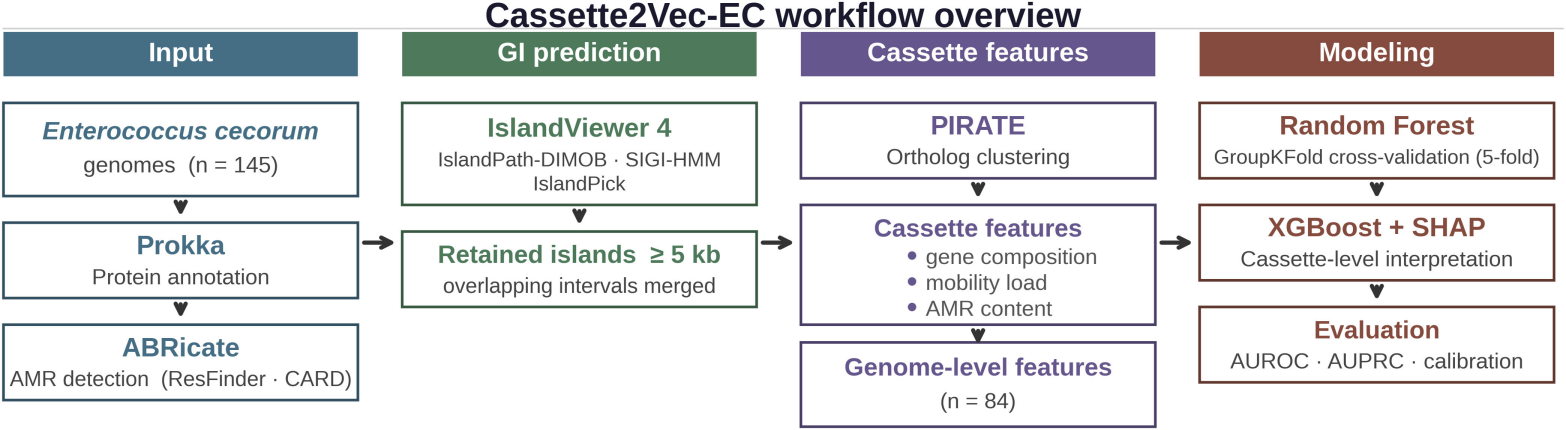
Cassette2Vec-EC workflow overview. Genomes → GI prediction and annotation (Prokka, IslandViewer, ABRicate, PIRATE) → cassette construction and vectorization → genome-grouped machine learning (Random Forest / XGBoost) → SHAP-based interpretation. GI, genomic island; ML, machine learning; SHAP, SHapley Additive exPlanations.

We analyzed a curated *E. cecorum* cohort (145 genomes; 95 commensal, 50 pathogenic after quality control; genome accessions and metadata are listed in Supplementary Data 1, which is deposited in Zenodo (doi: 10.5281/zenodo.18529389) and accessible without restriction). All genomes were retrieved from publicly accessible NCBI databases; no new animal sampling or experimental procedures were performed in this study, and therefore no institutional animal ethics approval was required. Phenotype labels were assigned from sampling context metadata: “pathogenic” isolates were from clinical disease contexts spanning the principal manifestations of *E. cecorum* disease in broilers: vertebral osteomyelitis and spondylitis presenting as posterior paresis (“kinky back”; Borst et al., 2015), frank lameness from outbreak investigations, and systemic/sepsis cases (Rhoads et al., 2024); specific anatomical isolation sites and clinical presentations vary by contributing BioProject and are documented in the originating publications accessible via the assembly accessions in Supplementary Data 1. “Commensal” isolates were from healthy-bird surveillance. Labels are metadata-derived and surveillance-grade; prospective validation is required before operational deployment. Genomes were annotated with Prokka v1.14.6 (Seemann, 2014). Genomic islands were predicted using IslandViewer 4 (Bertelli et al., 2017) integrating IslandPath-DIMOB, SIGI-HMM, and IslandPick; islands ≥ 5 kb were retained. Overlapping GI intervals were merged to a non-redundant set (≥1 bp overlap). AMR loci were screened with ABRicate v1.0.1 (Seemann, 2020) against ResFinder (Bortolaia et al., 2020), and CARD (Alcock et al., 2020) (identity ≥ 75%, coverage ≥ 80%). Orthologous clustering used PIRATE v1.0.4 (Bayliss et al., 2019). Functional annotation used eggNOG-mapper v2 (Cantalapiedra et al., 2021). The workflow is illustrated in Figure 1.

### Cassette construction and feature engineering

Cassette units were defined as contiguous GI-associated gene runs within merged IslandViewer intervals (≥1 bp overlap), bounded by contig ends or non-GI gaps. Large islands were not artificially capped by gene count. Each cassette was encoded as a 17-feature per-cassette numeric vector according to a 20-column schema: 17 active numeric features (GI_flag, amr_hit, mobility_score, start, end, island_start, island_end, island_length, amr_pid, amr_pcov, is_virulence, is_AMR, integrase_flag, transposase_flag, recombinase_flag, GI_AMR_density, Mobility_Load) plus 3 reserved schema columns (eggNOG_class, pathway, cluster_id) that were not populated in v1 (see Supplementary Data 4). Genome-level summary features (84 features) were computed by aggregating per-cassette values using mean, maximum, and sum statistics across all cassettes and GI-cassette subsets per genome. The 84-feature genome-level summary matrix was used for all benchmark comparisons; the 17-feature per-cassette representation was used for the original XGBoost model and SHAP interpretation.

### Benchmark design and locked evaluation protocol

All benchmark models were evaluated on the identical 145-genome cohort using a single pre-locked 5-fold genome-grouped cross-validation file (common_145genome_cv5_fold_file.xlsx; locked before any analysis, as documented in the Zenodo archive doi: 10.5281/zenodo.18529389, v1.0.0, deposited February 2026). Upstream cassette-construction files were generated from a broader 150-genome pre-QC set; all benchmark analyses reported here use the final QC-filtered 145-genome cohort. Each fold contained 29 genomes (10 pathogenic, 19 commensal). All benchmark models used Random Forest [scikit-learn v1.3.2 (Pedregosa et al., 2011); n_estimators = 500; random_state = 42; class_weight = ‘balanced’; n_jobs = 1 for reproducibility]. Preprocessing was fit on training folds and applied to held-out folds. Metrics: AUROC, area under the precision-recall curve (AUPRC), macro-averaged F1, and Brier calibration score (Brier, 1950). Fold-level performance differences were evaluated using paired comparisons across the five locked folds and interpreted cautiously because *n* = 5 folds provides limited statistical power. The final benchmark set comprised the Cassette2Vec-EC genome-summary RF model, a corrected AMR-content baseline, a GI-burden baseline, an assembly-quality baseline, a conservative GI-restricted gene product presence/absence proxy, a cassette + GI hybrid model, and the original XGBoost cassette-row model as an interpretability reference.

### External baseline construction

Four external baselines used the same cohort, fold file, and RF classifier where compatible: (i) assembly quality (contig count; 1 feature); (ii) GI burden summaries (15 per-genome island statistics; gi_burden_matrix_fixed_145genomes.xlsx); (iii) corrected AMR gene content (43 features from the corrected benchmark workbook AMR_Matrix_FIXED sheet, replacing an encoding error in which amr_list_entry_count_raw was set to zero for all 50 pathogenic genomes, producing artifactual perfect class separation; corrected values confirmed against raw ABRicate CSV files); and (iv) a conservative GI-restricted gene product presence/absence proxy (484 GI-localized product-family features; 144 matched genomes), used as an interim inventory-style comparator because a valid joint-run PIRATE GPA matrix was not available. Separate commensal and pathogenic PIRATE runs were not used as a full GPA baseline because PIRATE defines ortholog families within each run, making cross-class merged matrices non-comparable and prone to run-identity leakage. All benchmark feature matrices were regenerated and cross-checked against the final deposited analysis files where available.

### Fragmentation robustness analyses

Four analyses: (i) contig count added as 85th covariate to the 84-feature cassette RF model; (ii) ≤100 contigs subset (*n* = 123; 28 pathogenic); (iii) ≤50 contigs subset (*n* = 91; 21 pathogenic, 70 commensal); (iv) fragmentation-matched subset retaining all commensal genomes (*n* = 95) plus only pathogenic genomes with ≤ 50 contigs (*n* = 21; equalizing mean contig counts: pathogenic 32 vs. commensal 33).

### Cross-project validation

Leave-one-BioProject-out validation was used as the most stringent estimate of transportability to new surveillance contexts: the RF model was trained on 20 BioProjects (*n* = 92) and tested on held-out PRJNA1050746 (*n* = 53; 16 pathogenic, 37 commensal). Identical RF parameters were used. This analysis is independent of the locked fold protocol and should be interpreted as a deployment-oriented stress test rather than a replacement for within-cohort cross-validation.

### Model interpretability and mobilome signature

SHapley Additive exPlanations values (Lundberg and Lee, 2017) were computed at the cassette-row level using the XGBoost cassette-row model (which provides cassette-level granularity not available from the genome-level RF model). Genome-level explanations were derived by aggregating cassette-level SHAP values within each genome. The 20-gene non-AMR mobilome signature was derived using training-only gene enrichment per outer fold (log_2_ odds ratio of GI-localized presence; pathogenic vs. commensal). Stability was assessed as selection frequency across five outer training splits (Supplementary Data 5).

### Model configurations

Two complementary configurations are reported. (i) RF cassette-summary model (primary benchmark): Random Forest on 84 per-genome cassette aggregation features; used for all Tables 1–3 comparisons because it uses the same classifier as all external baselines, enabling directly comparable benchmarking. AUROC 0.918 ± 0.067. (ii) XGBoost cassette-row model (original implementation): XGBoost (Chen and Guestrin, 2016) trained on per-cassette feature rows with genome-level probability aggregation; GitHub v1.0.0; used for SHAP interpretation at cassette granularity. AUROC 0.874 ± 0.077.

**TABLE 1:** Benchmark comparison under identical 5-fold genome-grouped cross-validation (*n* = 145; locked fold file; RF *n* = 500; seed = 42; class_weight = ‘balanced’; n_jobs = 1; unless noted).

**Method**	**Feature basis**	**Feature count (*N*)**	**AUROC ± SD**	**AUPRC ± SD**	**Brier**	**Notes**
Cassette2Vec-EC ★	84 genome-level cassette summary features	84	**0.918 ± 0.067**	0.906 ± 0.061	0.102	RF *n* = 500; primary model
AMR gene content (corrected)	43 ABRicate-derived features (encoding bug corrected)	43	0.906 ± 0.044	0.879 ± 0.055	0.100	RF *n* = 500; independently reproduced
GI-restricted gene product P/A proxy§	484 GI-localized product-family presence/absence features	484	0.887 ± 0.083	0.855	0.134	Interim inventory-style comparator; not full joint PIRATE GPA
GI burden summaries	15 per-genome island statistics	15	0.791 ± 0.050	0.705 ± 0.059	0.172	RF *n* = 500; reproduced
Assembly quality only	Contig count (fragmentation proxy)	1	0.743 ± 0.015	0.739 ± 0.006	0.197	RF *n* = 500; confound check
Cassette + GI hybrid†	Cassette + GI burden (99 features)	99	0.915 ± 0.062	0.901 ± 0.059	0.104	RF *n* = 500; hybrid reference
XGBoost cassette-row model‡	17 per-cassette features; genome-level aggregation	17	0.874 ± 0.077	0.872 ± 0.063	0.108	XGBoost v2.0.3; original implementation

★Primary result. §GI-restricted gene product presence/absence proxy is an interim inventory-style comparator and not a full joint-run PIRATE GPA baseline. †Hybrid reference model (same RF); per-fold breakdown unavailable. see Supplementary Data 2 note. ‡ XGBoost v2.0.3 original implementation; genome-level probability aggregation. Brier = calibration score (lower is better). Fold-level paired comparisons: Cassette vs. AMR, mean AUROC Δ = +0.012, *p* = 0.745; Cassette vs. GI burden, Δ = +0.127, *p* = 0.026; Cassette vs. assembly quality, Δ = +0.175, *p* = 0.008. *P*-values are reported descriptively and interpreted cautiously because *n* = 5 folds. Bold values indicate the primary Cassette2Vec-EC model result.

**TABLE 2:** Cross-project leave-one-BioProject-out validation (train *n* = 92, test PRJNA1050746 *n* = 53; 16 pathogenic, 37 commensal; RF *n* = 500, seed = 42, n_jobs = 1).

**Model**	**AUROC (held-out project)**	**Delta vs. cassette**	**Interpretation**
Cassette2Vec-EC (84 feat)	0.694	–	Highest observed cross-project AUROC
AMR gene content (43 feat)	0.663	−0.031	Closest to cassette cross-project
GI burden (15 feat)	0.405	−0.289	GI burden fails cross-project
Assembly quality only (1 feat)	0.314	−0.380	Near-chance; completely fails

**TABLE 3:** Cassette-associated signal persists after controlling for assembly fragmentation analysis.

**Subset**	***N* (path/comm)**	**Path mean contigs**	**Cassette AUROC**	**AMR AUROC**	**Contig-only AUROC**
Full cohort (primary)	145 (50/95)	109	0.918 ± 0.067	0.906 ± 0.044	0.743 ± 0.015
≤100 contigs	123 (28/95)	42	0.844 ± 0.153	0.931 ± 0.061	0.525 ± 0.110
≤50 contigs ★ (high quality)	91 (21/70)	32	0.827 ± 0.148	0.915 ± 0.075	0.628 ± 0.219
Frag-matched (path ≤ 50 ctg)	116 (21/95)	32	0.838 ± 0.115	0.888 ± 0.109	0.742 ± 0.160
Cassette + contig covariate (ctrl)	145 (50/95)	109	0.916 (Δ = −0.002)	–	–

★≤50-contig subset is the primary fragmentation-controlled analysis. AUROC ± SD across quality-filtered and confound-controlled subsets. Path, pathogenic; Comm, commensal. All models: RF *n* = 500, seed = 42, n_jobs = 1. Covariate control row: cassette features + contig count as 85th feature.

## Results

**FIGURE 2:**
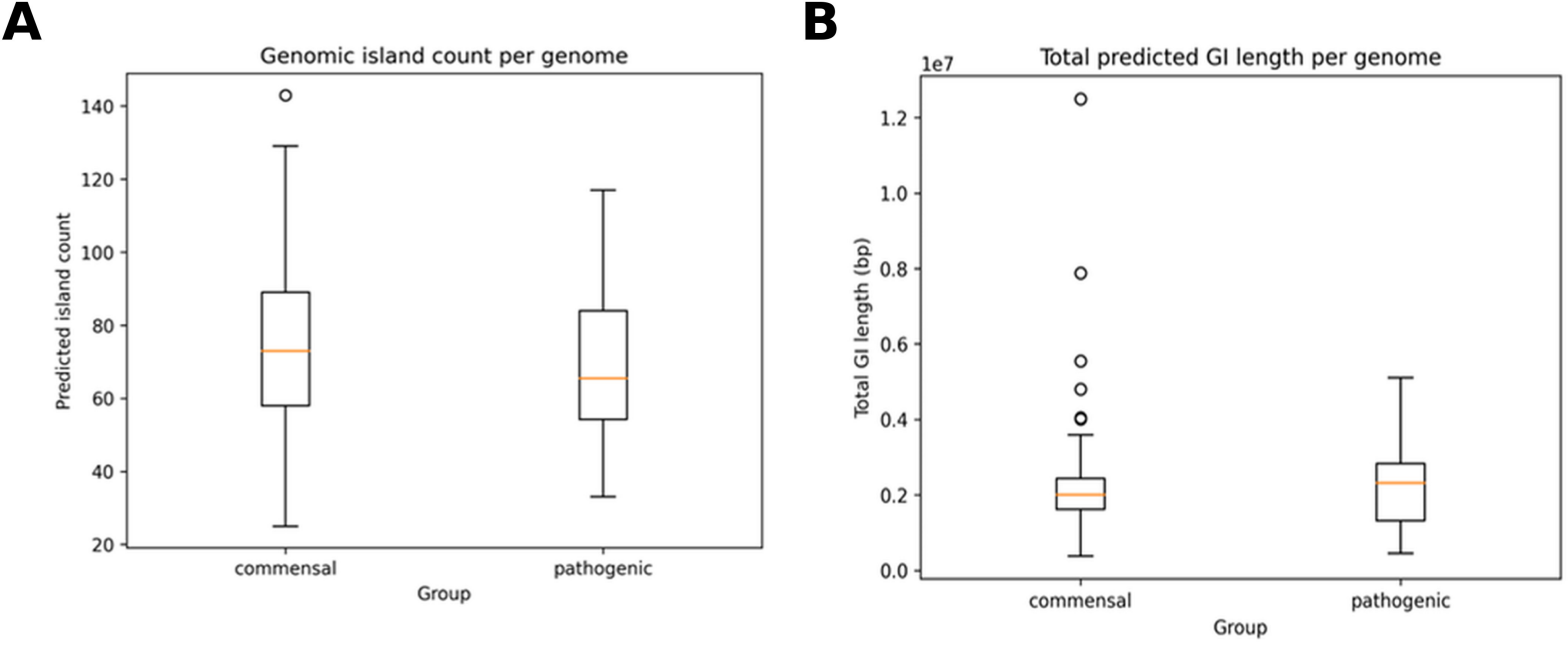
Genomic island (GI) burden does not separate commensal and pathogenic classes. **(A)** Predicted GI counts per genome. **(B)** Total predicted GI length per genome. *n* = 145 genomes (95 commensal, 50 pathogenic). Box plots show median and interquartile range; individual points overlaid. Two-sided Wilcoxon rank-sum test *p*-values: GI count *p* = 0.244; total GI length *p* = 0.388.

**FIGURE 3:**
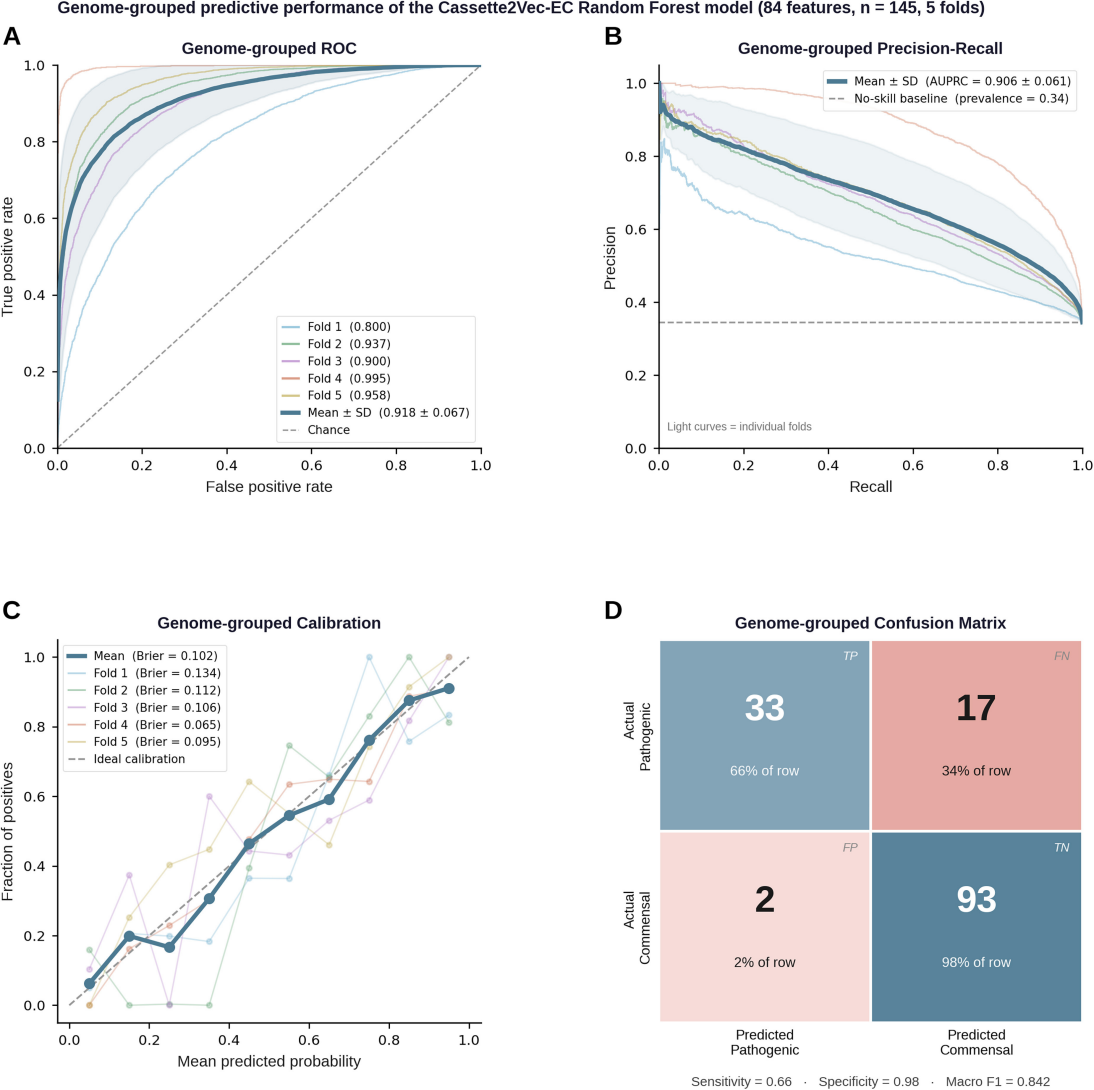
Genome-grouped predictive performance of the Cassette2Vec-EC Random Forest cassette-summary model (84 features, *n* = 145, 5-folds). **(A)** ROC curves across GroupKFold-by-genome folds (mean ± SD). **(B)** Precision-recall curves across folds. **(C)** Binned calibration curves (10 bins per fold). **(D)** Genome-level confusion matrix at threshold 0.5. All panels: 5-fold genome-grouped cross-validation; approximately 10 pathogenic genomes per held-out fold.

**FIGURE 4:**
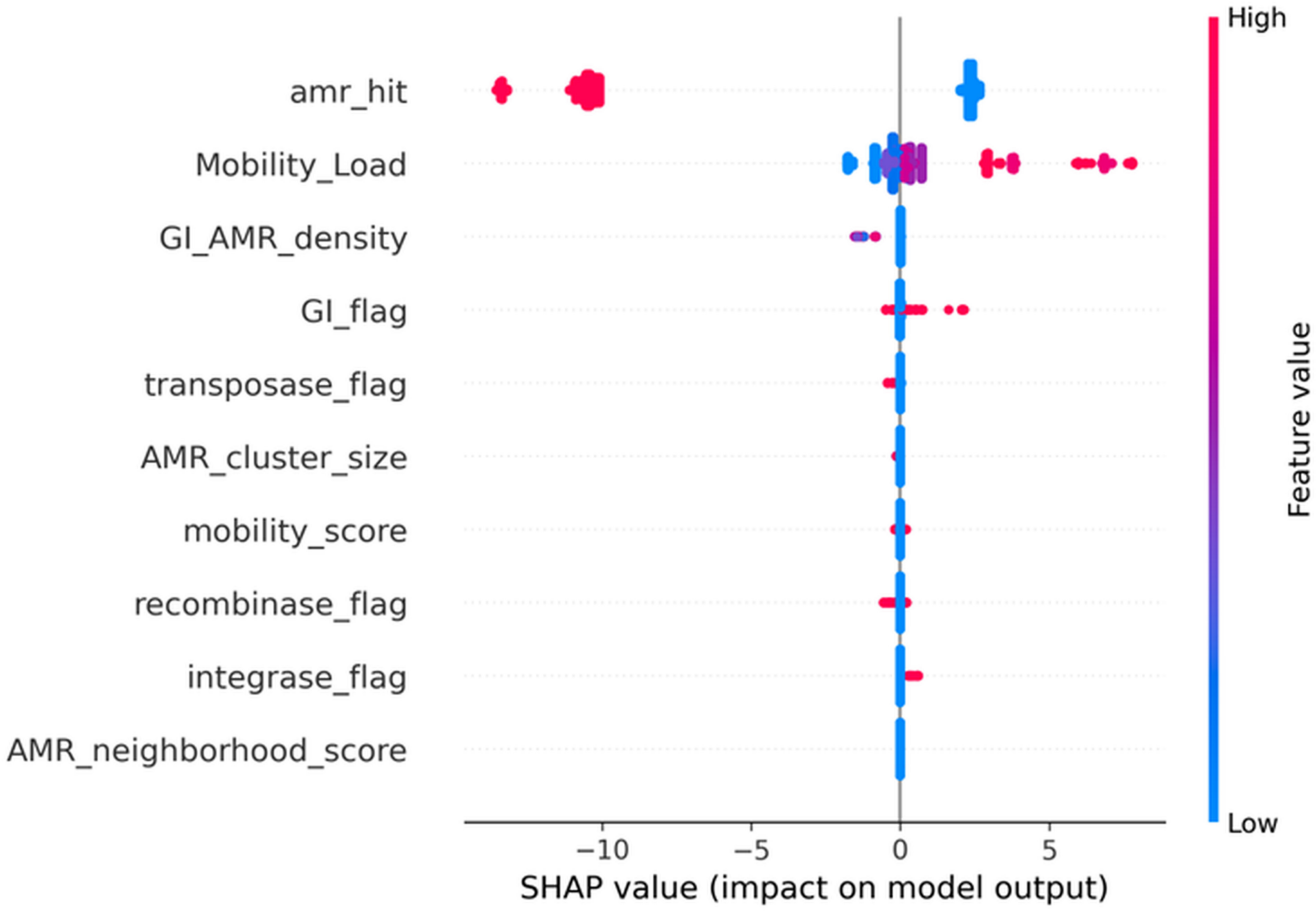
Model explainability for the XGBoost cassette-row implementation (original implementation; used for cassette-level SHAP interpretation only). SHAP summary (beeswarm) plot showing the top cassette- and context-level features contributing to the XGBoost cassette-row model output under genome-grouped evaluation. The primary benchmark comparisons in Tables 1–3 use the Random Forest cassette-summary model; this Figure provides cassette-level mechanistic interpretation not available from the genome-summary RF model. Points represent per-cassette SHAP values (impact on model prediction), with color indicating feature value (blue, low; red, high). Positive SHAP values increase the predicted probability of the Pathogenic class; negative values decrease it.

Results are organized in three tiers. Primary analysis: the RF cassette-summary model benchmarked against AMR content, GI burden, assembly quality, and the GI-restricted gene product presence/absence proxy under 5-fold genome-grouped cross-validation (Figures 2, 3 and Tables 1, 2). Secondary analyses: fragmentation robustness analyses confirming that cassette signal is not primarily driven by assembly quality differences (Table 3), and leave-one-BioProject-out cross-project validation as a deployment-oriented stress test. Exploratory analyses: SHAP feature attribution identifying candidate discriminant modules (Figure 4), and the non-AMR mobilome signature as a hypothesis-generating gene set for future experimental work.

### Genomic-island burden alone does not distinguish pathogenic from commensal lineages

We first tested whether simple genomic-island burden separated classes in our 145-genome cohort of *E. cecorum* genomes from poultry production systems (95 commensal, 50 pathogenic; Supplementary Data 1) and found substantial overlap in both GI count and total GI length between pathogenic and commensal genomes (Wilcoxon rank-sum: GI count *p* = 0.244, Cliff’s δ = −0.117; total GI length *p* = 0.388, Cliff’s δ = 0.088; Figure 2), confirming that univariate island burden does not separate pathogenic from commensal genomes. Pathogenic assemblies were substantially fragmented on average (mean contigs 109 vs. 33; 22 pathogenic genomes exceeded 100 contigs, maximum 428, versus none for commensals), motivating the fragmentation analyses below.

### GI-anchored cassette architecture provides interpretable classification signal comparable to AMR content and outperforms structural baselines

We next evaluated whether cassette organization captured signal beyond burden-only summaries. The 84-feature cassette-summary RF model achieved AUROC 0.918 ± 0.067 (AUPRC 0.906 ± 0.061; Brier 0.102; Figure 3), outperforming GI burden summaries (AUROC 0.791 ± 0.050; gap +0.127) and assembly quality alone (0.743 ± 0.015; gap +0.175) under identical cohort, fold assignments, and metrics (Table 1). Fold-level paired comparisons are consistent with a cassette advantage over GI burden (mean AUROC difference +0.127; paired *t*-test *p* = 0.026) and assembly quality (mean difference +0.175; *p* = 0.008); all fold-level comparisons should be treated as descriptive statistics rather than confirmatory tests, as *n* = 5 folds provides limited statistical power. The corrected AMR gene content baseline achieved AUROC 0.906 ± 0.044, which was statistically indistinguishable from the cassette model at fold level (mean difference +0.012; paired *t*-test *p* = 0.745). A conservative GI-restricted gene product presence/absence proxy achieved AUROC 0.887 ± 0.083 (AUPRC 0.855; Brier 0.134), providing an interim inventory-style comparator until a full joint-run PIRATE GPA matrix is generated. A hybrid model adding GI burden to cassette features (99 features, AUROC 0.915 ± 0.062) provided no improvement over cassette features alone (Δ = −0.003 AUROC, −0.005 AUPRC), confirming no incremental gain from adding GI burden features to the cassette representation.

The AMR and inventory-style baseline comparisons require honest interpretation. In the full cohort, cassette (0.918) and AMR content (0.906) are approximately equal in AUROC, and the GI-restricted presence/absence proxy is only modestly lower (0.887). For a user requiring only a genome risk score without mechanistic interpretation, AMR gene content is therefore a competitive quick proxy. What AMR content and flat inventory features cannot provide is cassette-level explanation: which specific island-anchored gene neighborhoods drive a classification, at which genomic coordinates, in which mobility context, with what co-cargo. Cassette-level SHAP analysis (see below) shows that the discriminating signal localizes to co-organization of AMR cargo with mobility machinery within GI-anchored modules, not AMR presence alone.

For reference, the original XGBoost cassette-row model (17 per-cassette features; genome-level probability aggregation; GitHub v1.0.0) achieved AUROC 0.874 ± 0.077, lower than the RF cassette-summary model on this dataset, consistent with genome-level feature aggregation reducing within-genome redundancy for this cohort size.

### Cross-project validation provides a more stringent deployment-oriented estimate

To estimate performance in a more realistic surveillance setting, we trained on 20 BioProjects and tested on a fully held-out BioProject (PRJNA1050746; *n* = 53; 16 pathogenic, 37 commensal). Cassette features retained the highest observed AUROC among the tested models (0.694; margin over AMR baseline: +0.031), with AMR content closely behind (0.663), while GI burden (0.405) and assembly quality (0.314) largely fail (Table 2). Cross-project validation provides a more stringent estimate of generalization performance than within-cohort cross-validation and suggests the model captures both biological signal and project-specific structure. The performance drop from standard CV (0.918) to cross-project validation (0.694) is substantial and reflects project-correlated structure: genomes from the same BioProject share sequencing platform, isolation context, and phylogenetic background. We interpret this transparently: the cross-project AUROC of 0.694 represents the best current estimate of real-world performance on genomes from new surveillance contexts and should be the primary metric for deployment-oriented performance estimates. Cassette and AMR content perform similarly cross-project (0.694 vs. 0.663), consistent with both methods partially capturing project-correlated rather than purely biology-specific signal.

### Cassette-associated signal persists after controlling for assembly fragmentation

Because pathogenic assemblies were more fragmented on average than commensal assemblies, we performed four analyses to determine whether cassette performance reflected assembly quality rather than biological signal (Table 3). These analyses establish that the cassette model’s performance is not primarily driven by assembly quality differences. First, adding contig count as an 85th covariate changed cassette AUROC by only −0.002 (0.918 → 0.916), confirming that fragmentation provides no additional information beyond the cassette features. Second, in the ≤50-contig high-quality subset (*n* = 91; 21 pathogenic), the contig-count baseline fell from 0.743 to 0.628 while cassette AUROC was 0.827, demonstrating that the assembly quality signal weakens as fragmentation differences between classes are reduced. Third, in the fragmentation-matched subset (*n* = 116; mean contig counts equalized at 32 vs. 33), cassette AUROC was 0.838, substantially above the contig-count baseline (0.742). Fourth, the AMR gene content baseline was stable or higher in quality-filtered subsets (≤50 contigs: 0.915), consistent with AMR gene detection being largely independent of assembly contiguity. Together, these analyses support the interpretation that cassette features capture biologically consistent signal rather than a pure technical fragmentation artifact. We note that cassette construction depends on coordinate-based contiguity and fragmented assemblies may underestimate cassette sizes, potentially attenuating rather than inflating the cassette signal.

### SHAP localizes predictive signal to GI-associated AMR and mobility modules

SHapley Additive exPlanations analysis of the XGBoost cassette-row model (Figure 4) localized discriminant signal to GI-associated modules enriched for AMR cargo, mobility load, and GI AMR density. At the feature level, the top discriminating features included amr_hit (direct AMR cargo indicator), Mobility_Load (co-localized transposase/integrase/recombinase count), and GI_AMR_density (AMR gene density within the island) as the top discriminating features. Mobility marker flags (transposase_flag, integrase_flag, recombinase_flag) contributed consistent positive signal, while GI_AMR_density showed bidirectional effects, indicating that AMR gene density within island context modulates rather than uniformly increases predicted risk. This pattern supports the cassette framing: pathogenic lineages are distinguished by co-localization of AMR cargo with mobility machinery within GI-anchored cassettes, not solely by AMR gene presence.

These SHAP-identified feature associations are descriptive of model behavior and should not be interpreted as validated biological mechanisms; experimental confirmation of these candidate modules as pathogenicity determinants is required before mechanistic conclusions can be drawn.

The 20-gene non-AMR mobilome signature (Supplementary Data 5) identifies recurrent GI-localized modules: specific xerc/xerd paralogs (xerc_3, xerd_2 at 5/5; xerc_5, xerc_6 at 4/5; see Supplementary Data 5), CRISPR-associated Cas2 (4/5), plasmid replication proteins RepB family (3–4/5), and single-stranded DNA-binding proteins Ssb (3/5). These non-AMR loci span mobile-element recombination, plasmid maintenance, and DNA repair functions, consistent with, but not confirmatory of, the hypothesis that pathogenic EC lineages carry recurrent horizontally acquired modules including both resistance cargo and the mobility/maintenance machinery required for transfer and stable integration.

### Comparative AMR prevalence

Comparative AMR gene prevalence analysis (Supplementary Figure 1) confirms that pathogenic genomes show higher prevalence of tetL/tetL2 (pathogenic 82% vs. commensal 46%), mel/mefA family macrolide resistance genes (40%–44% vs. 22%–26%), and lincosamide resistance loci. Representative cassette architectures from a closed reference genome (Supplementary Figure 2) illustrate how resistance genes (mefA/msrD/ermG; tetM/tetL) are physically co-organized with mobility markers within GI intervals — the structured biological signal that cassette features encode and flat gene-presence lists discard.

## Discussion

A central uncertainty underlying all results in this study is the metadata-derived nature of the phenotype labels. “Pathogenic” isolates were defined as those recovered from clinical disease contexts (osteomyelitis, lameness, sepsis outbreaks), while “commensal” isolates were from healthy-bird surveillance. This classification is practical and consistent with the published *E. cecorum* literature, but it does not constitute true phenotypic validation: an isolate from a diseased bird may be a secondary colonizer or bystander rather than a primary pathogen, and an isolate from a healthy bird may carry unexpressed pathogenic potential. All model performance metrics, SHAP attributions, and biological interpretations reported here are conditioned on this labeling scheme. The model learns to separate surveillance contexts as much as it learns to separate biological phenotypes. This caveat should be borne in mind throughout the interpretation of all results.

### Summary of principal findings

This study shows that, in poultry-associated *E. cecorum*, genomic-island-anchored cassette organization contains predictive signal for pathogenic lineage classification beyond genomic-island burden and assembly-quality proxies. In the full cohort, cassette features performed similarly to a corrected AMR gene-content baseline and modestly above a conservative GI-restricted gene product presence/absence proxy, while retaining the advantage of module-level interpretability. The main contribution of this study is therefore not the identification of pathogenic clones or mobile-element-associated features per se, nor a claim that cassette features dominate all inventory-based alternatives, but the demonstration that accessory-genome organization can be encoded as an informative and interpretable genomic representation in this organism. The 0.127-point AUROC advantage over GI burden (0.918 vs. 0.791) is meaningful because island counts and lengths alone are weak classifiers, consistent with the finding that GI count and total GI length do not separate classes in univariate analysis.

Across evaluation settings, cassette features outperform GI burden in the full cohort, outperform assembly quality across all tested settings, and remain biologically informative under quality-controlled conditions, although AMR content performs better in the stricter quality-filtered subsets (e.g. ≤50 contigs: 0.915 vs. 0.827). Cassette features uniquely provide module-level interpretable explanations that AMR inventories and flat gene-product presence/absence proxies cannot supply. A full joint-run pangenome GPA benchmark remains an important next analysis, but available separate PIRATE outputs could not be validly merged without introducing run-identity leakage.

### Relationship to AMR gene content

The corrected AMR baseline (AUROC 0.906) performed similarly to the cassette model (0.918) in the full cohort, indicating that AMR inventories remain a strong surveillance-oriented proxy in this dataset. Fold-level comparison did not support a statistically reliable AUROC difference between cassette and AMR models (paired *t*-test *p* = 0.745), so we interpret these models as comparable in rank-order performance. The value added by the cassette representation is therefore not that it fully replaces AMR-based prediction, but that it places predictive signal into genomic context by identifying which GI-associated neighborhoods, mobility features, and co-localized modules contribute to classification. This is directly supported by SHAP, in which discriminant signal localizes to GI_AMR_density and Mobility_Load rather than to amr_hit alone. Under quality-controlled conditions (≤50 contigs), AMR content performed better than cassette features (0.915 vs. 0.827), consistent with AMR gene detection being less sensitive to assembly fragmentation than coordinate-dependent cassette construction. Cross-project performance was also similar for cassette and AMR representations (0.694 vs. 0.663), suggesting that both capture biologically consistent signal while remaining partly influenced by project-specific structure.

### Connection to prior *E. cecorum* genomics

Prior EC genomics studies have characterized divergent genomic features of pathogenic lineages (Borst et al., 2015), documented intercontinental spread of clinical clones (Laurentie et al., 2023), identified elevated AMR gene carriage and GI content in outbreak-associated isolates (Jackson et al., 2015; Huang et al., 2024), and reported molecular genomic heterogeneity among *E. cecorum* isolates from broiler sepsis outbreaks (Rhoads et al., 2024). These studies established that pathogenic *E. cecorum* genomes carry distinctive mobile genetic element repertoires, but did not develop or benchmark predictive classifiers capable of assigning risk scores to new genomes. Our results extend that literature by suggesting that the physical organization of AMR and mobility features within GI-associated modules carries useful signal beyond simple inventory-based summaries. The SHAP-identified mobilome signature tyrosine recombinases of the XerC/D family, plasmid replication proteins, and CRISPR-associated Cas2 is consistent with the mobile element-enriched genomic islands previously described in pathogenic EC by Borst et al. (2015), Laurentie et al. (2023), Rhoads et al. (2024), providing convergent biological support for the feature set.

The 145-genome cohort reflects the current practical ceiling of publicly available, phenotypically annotated EC genomes from poultry production systems. EC remains an emerging pathogen with substantially fewer sequenced genomes than common enteric pathogens; the largest prior comparative EC genomics study of pathogenic versus commensal isolates used a similar order-of-magnitude cohort (Borst et al., 2015; Laurentie et al., 2023). Genome-grouped cross-validation with locked fold assignments directly addresses the small-cohort challenge by preventing within-genome inflation and providing honest per-fold variance estimates. The cross-project validation (AUROC 0.694 on a held-out BioProject of 53 genomes) is arguably more informative than the within-study estimate for assessing deployability, and the variance reported across folds provides the uncertainty context that AUROC point estimates alone cannot convey. Expansion of this cohort through prospective sequencing and collaborative phenotyping is the primary path to increasing classifier confidence and generalizability.

### Cross-project performance and calibration

The lower leave-one-BioProject-out AUROC (0.694) indicates that transportability to new surveillance contexts is more limited than the within-cohort cross-validation results alone would suggest. This reflects a combination of lineage structure, project-specific sampling effects, and the broader challenge of transferring predictive models across surveillance contexts. For that reason, the present results should be interpreted as evidence that cassette organization is biologically informative in this cohort, but not yet as proof of deployment-ready generalization. The cross-project AUROC of 0.694 represents our current best estimate of real-world performance on genomes from new surveillance contexts and should be the primary metric for deployment-oriented performance estimates. Cassette and AMR content perform similarly in the cross-project setting (0.694 vs. 0.663), consistent with both methods partly capturing project-correlated rather than purely biology-specific signal. Prospective validation on additional independent collections is required before deployment claims can be made.

GI burden summaries and assembly quality baselines are weaker classifiers (AUROC 0.791, 0.743) with poorer Brier scores (0.172, 0.197) compared to the cassette model (Brier 0.102) and AMR content (Brier 0.100). For surveillance applications where probability outputs are used to prioritize isolates for follow-up, well-calibrated risk scores enable coherent threshold selection and uncertainty quantification. The cassette model and AMR content both provide better-calibrated probabilities than structural baselines across the full cohort.

Practical sensitivity and threshold considerations: At the default classification threshold of 0.5, the model correctly identified 33 of 50 pathogenic genomes (sensitivity = 0.66) while misclassifying 17 as commensal (false-negative rate = 0.34). For surveillance applications where missing a pathogenic genome carries higher practical cost than a false positive, this false-negative rate may be unacceptably high. Users should explore threshold-adjusted classification using the calibrated probability outputs (Brier score 0.102): lowering the threshold from 0.5 to 0.3 or 0.2 increases sensitivity at the cost of specificity, and the precision-recall curves (Figure 3B) provide the full tradeoff curve for threshold selection. Operational deployment should include explicit threshold optimization for the intended surveillance context.

### Future directions

This study has several limitations. First, the cohort is restricted to *E. cecorum* from poultry production systems; generalization to other Enterococcus species, hosts, or geographic regions remains to be established. Second, phenotype labels are metadata-derived and inherently imperfect; isolates from disease contexts may include bystanders and commensal isolates may harbor unexpressed pathogenic potential. Third, external validation on a fully independent cohort was not performed; prospective evaluation in independent collections is ongoing. Fourth, cross-project AUROC of 0.694 should be treated as the more realistic deployment performance estimate, not the within-study 0.918. Fifth, AMR gene content achieves comparable full-cohort AUROC and outperforms cassette in quality-filtered subsets, a finding we report honestly; the cassette advantage is clearer in calibration, structural interpretability, and SHAP attribution than in rank-order AUROC alone. Sixth, a full joint-run pangenome GPA baseline was not available in the current package; separate commensal and pathogenic PIRATE runs cannot be merged into a valid GPA matrix because ortholog-family definitions are run-specific. Seventh, SHAP features are associative and require experimental validation. Eighth, cassette construction is coordinate-based and sensitive to assembly contiguity; long-read sequencing will enable direct validation of GI boundary definitions.

The highest priority next analysis is generation of a single joint PIRATE pangenome run across all 145 genomes under the same locked evaluation protocol, which is the critical benchmark needed to determine whether cassette architecture provides interpretive value beyond a well-constructed gene inventory. Future work should further focus on applying lineage-aware or geographically independent validation, and testing whether recurrent high-value cassette junctions can be confirmed as stable surveillance markers.

## Conclusion

*E. cecorum* genomic island cassette architecture captures pathogenicity-associated signal beyond island burden and assembly-quality proxies and performs similarly to AMR gene content while retaining genomic-context interpretability. The predictive signal localizes to GI-associated modules enriched for AMR and mobility features, indicating that accessory-genome organization contributes meaningfully to pathogenic lineage discrimination in this dataset. These findings support cassette-based genomic representation as a useful surveillance-oriented approach for *E. cecorum*, while also indicating that broader deployment will require stronger external validation.

## Data Availability

The original contributions presented in this study are included in the article/Supplementary material, further inquiries can be directed to the corresponding author.

## Supplementary material

Supplementary Figure 1 Comparative antimicrobial resistance gene prevalence across commensal and pathogenic Enterococcus cecorum genomes. Bars show prevalence of the top antimicrobial resistance genes detected in each cohort class, highlighting higher prevalence of selected tetracycline, macrolide, and lincosamide resistance loci among pathogenic genomes.

Supplementary Figure 2 Representative genomic-island-anchored cassette architectures in a closed Enterococcus cecorum reference genome. Gene-level diagrams illustrate the physical co-organization of antimicrobial resistance loci, including mefA/msrD/ermG and tetM/tetL regions, with mobility-associated genes within predicted genomic-island intervals.

## References

[B1] Alcock B. P. Raphenya A. R. Lau T. T. Y. Sharma A. N. Butkiewicz M. Bobyr W. et al. (2020). CARD 2020: Antibiotic resistome surveillance with the comprehensive antibiotic resistance database. *Nucleic Acids Res.* 48 D517–D525. 31665441 10.1093/nar/gkz935 PMC7145624

[B2] Bayliss S. C. Thorpe H. A. Coyle N. M. Sheppard S. K. Feil E. J. (2019). PIRATE: A fast and scalable pangenomics toolbox for clustering diverged orthologues in bacteria. *Bioinformatics* 35 4207–4209. 10.1093/bioinformatics/btz190 31598686 PMC6785682

[B3] Bertelli C. Laird M. R. Williams K. P. Lau B. Y. Hoad G. Winsor G. L. et al. (2017). IslandViewer 4: Expanded prediction and visualization of genomic islands for larger-scale datasets. *Nucleic Acids Res.* 45 W30–W35. 10.1093/nar/gkx343 28472413 PMC5570257

[B4] Boerlin P. Nicholson V. Brash M. Slavic D. Boyen F. Sanei B. et al. (2012). Diversity of *Enterococcus cecorum* from chickens. *Vet. Microbiol.* 157 405–411. 10.1016/j.vetmic.2012.01.001 22266160

[B5] Borst L. B. Suyemoto M. M. Barnes H. J. (2017). Pathogenesis of enterococcal spondylitis caused by *Enterococcus cecorum* in broiler chickens. *Vet. Pathol.* 54 61–73. 10.1177/0300985816658098 27511310

[B6] Borst L. B. Suyemoto M. M. Robbins K. M. Lyman R. L. Martin M. P. Barnes H. J. (2012). Molecular epidemiology of *Enterococcus cecorum* isolates recovered from enterococcal spondylitis outbreaks in the southeastern United States. *Avian Pathol.* 41 479–485. 10.1080/03079457.2012.718070 22978557

[B7] Borst L. B. Suyemoto M. M. Scholl E. H. Fuller F. J. Barnes H. J. (2015). Comparative genomic analysis identifies divergent genomic features of pathogenic *Enterococcus cecorum*. *PLoS One* 10:e0121294. 10.1371/journal.pone.0121294 25860249 PMC4393107

[B8] Bortolaia V. Kaas R. S. Ruppe E. Roberts M. C. Schwarz S. Cattoir V. et al. (2020). ResFinder 4.0 for predictions of phenotypes from genotypes. *J. Antimicrob. Chemother.* 75 3491–3500. 10.1093/jac/dkaa345 32780112 PMC7662176

[B9] Brier G. W. (1950). Verification of forecasts expressed in terms of probability. *Mon. Weather Rev.* 78 1–3. 10.1175/1520-0493(1950)078<0001:VOFEIT>2.0.CO;2

[B10] Cantalapiedra C. P. Hernandez-Plaza A. Letunic I. Bork P. Huerta-Cepas J. (2021). eggNOG-mapper v2: Functional annotation, orthology assignments, and domain prediction at the metagenomic scale. *Mol. Biol. Evol.* 38 5825–5829. 10.1093/molbev/msab293 34597405 PMC8662613

[B11] Chen T. Guestrin C. (2016). “XGBoost: a scalable tree boosting system,” in *Proceedings of the 22nd ACM SIGKDD International Conference Knowledge. Discovery Data Mining*, (New York, NY: ACM), 785–794. 10.1145/2939672.2939785

[B12] De Herdt P. Defoort P. Vansteelant J. Swam H. Tanghe L. Van Goethem S. et al. (2009). *Enterococcus cecorum* osteomyelitis and arthritis in broiler chickens. *Vlaams Diergeneeskd. Tijdschr.* 78 44–48. 10.21825/vdt.87495

[B13] Dolka B. Czopowicz M. Chrobak-Chmiel D. Rzewuska M. Szeleszczuk P. (2016). Phenotypic and genotypic characterization of *Enterococcus cecorum* strains associated with infections in poultry. *BMC Vet. Res.* 12:129. 10.1186/s12917-016-0761-1 27350248 PMC4924287

[B14] Grund A. Rautenschlein S. Jung A. (2021). Tenacity of *Enterococcus cecorum* under different environmental conditions. *J. Appl. Microbiol.* 130 1494–1507. 10.1111/jam.14899 33064913

[B15] Higuita J. C. Arango M. Forga A. Cortes D. Graham D. (2025). An updated review of *Enterococcus cecorum* infections in poultry. *Avian Dis.* 68 404–411. 10.1637/aviandiseases-D-24-00098 40249579

[B16] Huang Y. Boyen F. Antonissen G. Vereecke N. Van Immerseel F. (2024). The genetic landscape of antimicrobial resistance genes in *Enterococcus cecorum* broiler isolates. *Antibiotics* 13:409. 10.3390/antibiotics13050409 38786138 PMC11117384

[B17] Jackson C. R. Kariyawasam S. Borst L. B. Frye J. G. Barrett J. B. Hiott L. M. et al. (2015). Antimicrobial resistance, virulence determinants and genetic profiles of clinical and nonclinical *Enterococcus cecorum* from poultry. *Lett. Appl. Microbiol.* 60 111–119. 10.1111/lam.12374 25470229

[B18] Jung A. Rautenschlein S. (2014). Comprehensive report of an *Enterococcus cecorum* infection in a broiler flock in Northern Germany. *BMC Vet. Res.* 10:311. 10.1186/s12917-014-0311-7 25539747 PMC4297365

[B19] Jung A. Chen L. R. Suyemoto M. M. Barnes H. J. Borst L. B. (2018). A review of *Enterococcus cecorum* infection in poultry. *Avian Dis.* 62 261–271. 10.1637/11825-030618-Review.1 30339512

[B20] Lagad R. R. Rafi S. Goswami A. (2026). Genomic-island cassette architecture enables interpretable pathogenic lineage classification outperforming structural baselines in poultry-associated *Enterococcus cecorum*. *bioRxiv* [Preprint] 10.64898/2026.02.21.707173 PMC13388253 42487707

[B21] Laurentie J. Loux V. Hennequet-Antier C. Achard C. S. Dheilly A. Nicolas P. et al. (2023). Comparative genome analysis of Enterococcus cecorum reveals intercontinental spread of a lineage of clinical poultry isolates. *mSphere* 8 e495–e422. 10.1128/msphere.00495-22 36794931 PMC10117131

[B22] Lundberg S. M. Lee S.-I. (2017). A unified approach to interpreting model predictions. *Adv. Neural Inf. Process. Syst.* 30 4765–4774. 10.48550/arXiv.1705.07874

[B23] Pedregosa F. Varoquaux G. Gramfort A. Michel V. Thirion B. Grisel O. et al. (2011). Scikit-learn: machine learning in Python. *J. Mach. Learn. Res.* 12 2825–2830.

[B24] Rhoads D. D. Pummill J. Alrubaye A. A. K. (2024). Molecular genomic analyses of *Enterococcus cecorum* from sepsis outbreaks in broilers. *Microorganisms* 12:250. 10.3390/microorganisms12020250 38399654 PMC10892122

[B25] Robbins K. M. Suyemoto M. M. Lyman R. L. Martin M. P. Barnes H. J. Borst L. B. (2012). An outbreak and source investigation of enterococcal spondylitis in broilers caused by *Enterococcus cecorum*. *Avian Dis.* 56 768–773. 10.1637/10253-052412-Case.1 23397855

[B26] Seemann T. (2014). Prokka: Rapid prokaryotic genome annotation. *Bioinformatics* 30 2068–2069. 10.1093/bioinformatics/btu153 24642063

[B27] Seemann T. (2020). *ABRicate: Mass Screening of Contigs for Antimicrobial Resistance or Virulence Genes. GitHub.* Available online at: https://github.com/tseemann/abricate (accessed June 22, 2026).

[B28] Sharma P. Gupta S. K. Barrett J. B. Hiott L. M. Woodley T. A. Kariyawasam S. et al. (2020). Comparison of antimicrobial resistance and pan-genome of clinical and non-clinical Enterococcus cecorum from poultry. *Foods* 9:686. 10.3390/foods9060686 32466367 PMC7353540

[B29] Siguier P. Gourbeyre E. Chandler M. (2014). Bacterial insertion sequences: Their genomic impact and diversity. *FEMS Microbiol. Rev.* 38 865–891. 10.1111/1574-6976.12067 24499397 PMC7190074

[B30] Souillard R. Laurentie J. Kempf I. Hellard G. Jouy E. Dheilly A. et al. (2022). Increasing incidence of Enterococcus-associated diseases in poultry in France over the past 15 years. *Vet. Microbiol.* 269:109426. 10.1016/j.vetmic.2022.109426 35526479

[B31] Wijetunge D. S. Dunn P. Wallner-Pendleton E. Lintner V. Lu H. Kariyawasam S. (2012). Fingerprinting of poultry isolates of Enterococcus cecorum using three molecular typing methods. *J. Vet. Diagn. Invest.* 24 1166–1171. 10.1177/1040638712463563 23104952

