# Supplementary figures and images for: Genomic-island cassette architecture provides interpretable signal for exploratory classification of poultry-associated Enterococcus cecorum lineages
Source: Front Microbiol. 2026 Jul 8;17:1882753. doi: 10.3389/fmicb.2026.1882753 (PMC13388253; doi:10.3389/fmicb.2026.1882753)

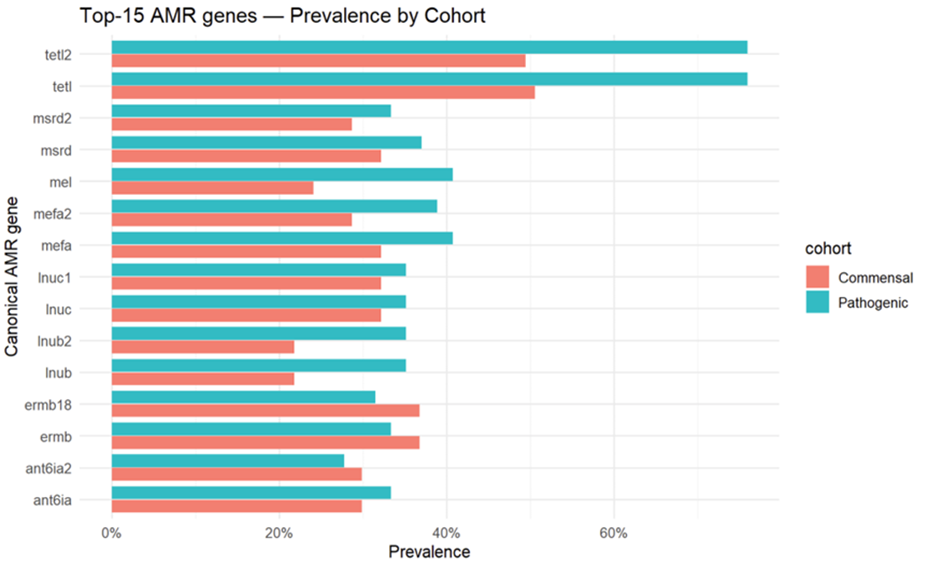

Supplement: Supplementary Figure 1 — Comparative antimicrobial resistance gene prevalence across commensal and pathogenic Enterococcus cecorum genomes. Bars show prevalence of the top antimicrobial resistance genes detected in each cohort class, highlighting higher prevalence of selected tetracycline, macrolide, and lincosamide resistance loci among pathogenic genomes. [file Image_1.tif]

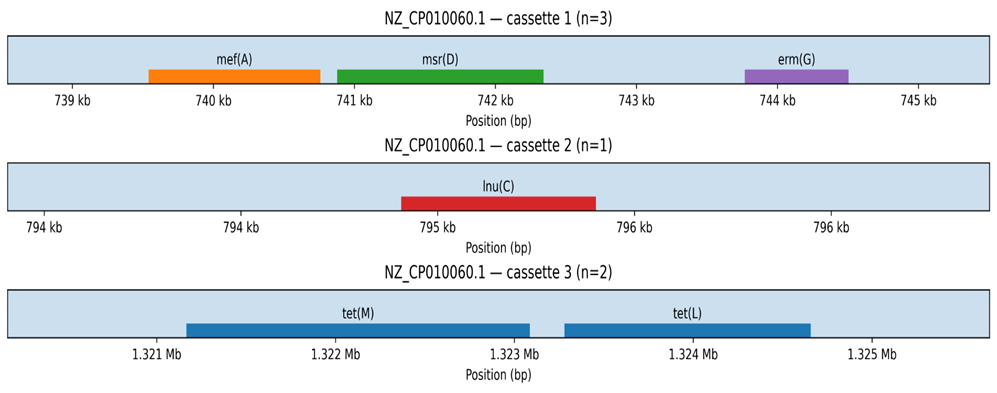

Supplement: Supplementary Figure 2 — Representative genomic-island-anchored cassette architectures in a closed Enterococcus cecorum reference genome. Gene-level diagrams illustrate the physical co-organization of antimicrobial resistance loci, including mefA/msrD/ermG and tetM/tetL regions, with mobility-associated genes within predicted genomic-island intervals. [file Image_2.tif]
